# Virus discharge and initial gastrointestinal involvement are inversely associated with circulating lymphocyte count in COVID-19

**DOI:** 10.7150/ijms.51672

**Published:** 2021-01-01

**Authors:** Wei Chen, Kenneth I. Zheng, Saiduo Liu, Chongyong Xu, Chao Xing, Zengpei Qiao

**Affiliations:** 1 Department of Radiology, the Second Affiliated Hospital of Wenzhou Medical University, Yuying Chilldren's Hospital, Wenzhou, China; 2 MAFLD Research Center, Department of Hepatology, the First Affiliated Hospital of Wenzhou Medical University, Wenzhou, China; 3 Department of Infectious Disease, the Sixth People's Hospital of Wenzhou, China; 4 Department of Laboratory Medicine, the Second Affiliated Hospital of Wenzhou Medical University, Yuying Children's Hospital, Wenzhou, China

**Keywords:** circulating lymphocyte count, COVID-19, gastrointestinal tract, CRP, stool, emergency

## Abstract

**Background:** It's reported SARS-CoV-2 could transmit via gastrointestinal tract, with or without pulmonary symptoms. However, as far as we know, there is no effective marker to predict the virus discharge in stool and initial gastrointestinal involvement of COVID-19 patients.

**Aims**: We aimed to investigate the likely biomarker predicting virus discharge in stool and initial gastrointestinal involvement of COVID-19, which may assist the clinicians in better preventing COVID-19 spread.

**Methods:** The patients complained of gastrointestinal symptoms, including vomiting, diarrhea, with or without respiratory symptoms, attending the Sixth People's Hospital of Wenzhou, and the Second Affiliated Hospital of Wenzhou Medical University, were screened by qRT-PCR for SARS-CoV-2. The confirmed COVID-19 patients, without any history of intaking contaminated food or water, were all enrolled to investigate the association between circulating lymphocyte count and virus discharge, initial gastrointestinal involvement.

**Results**: Seventy-six COVID-19 patients were included in the final analysis (mean age of 44.5 years, male 44.7%), with 24 (31.5%) complained of initial gastrointestinal symptoms. Significantly lower circulating lymphocyte count was found in the patients with positive results of qRT-PCR on stool (p = 0.012). Patients were divided into tertile groups by circulating lymphocyte count: lymphocyte ≤0.88*10^^9^/l ( n = 25 ), 0.88*10^^9^/l -1.2*10^^9^/l ( n = 28 ), and >1.2*10^^9^/l ( n = 23 ), respectively. When circulating lymphocyte count increased from 1st tertile to the 2nd and 3rd tertiles, the risk of initial gastrointestinal symptoms decreased by nearly 75% (OR = 0.25, 95% CI: 0.07, 0.98, p = 0.047), 83% (OR = 0.17, 95% CI: 0.05, 0.63, p = 0.008), after adjusting for likely confounders.

**Conclusions**: The circulating lymphocyte count is inversely associated with virus discharge in stool, and the risk of initial gastrointestinal involvement in COVID-19 patients.

## Introduction

The Coronavirus (CoVs) belongs to the subfamily of *Ortho-coronavirinae* in the family of *Coronaviridae* and the *Order Nidovirales*. In 2003, a SARS-CoV had caused the outbreak of severe acute respiratory syndrome 1. The virus responsible for the epidemic worldwide in late 2019, has been identified as novel coronavirus by World Health Organization (WHO), and termed as SARS-CoV-2, characterized as highly contagious and deadly. By the end of August 2020, more than 17 million cases infected with SARS-CoV-2, and more than 600,000 of deaths were confirmed worldwide, and the COVID-19 has been declared a pandemic by WHO.

Emerging evidences showed that gastrointestinal (GI) might be affected by SARS-CoV-2, though the virus is mainly identified transmitting through respiratory droplet 2. And furthermore, a COVID-19 patient could still discharge virus via GI tract even after pulmonary symptoms have resolved, and thus, those who got recovered from COVID-19 and tested negative on consecutive oropharyngeal swabs may not be considered to be safe 3. It is also evident that SARS-CoV-2 may initially affect the GI tract and with positivity of viral RNA in the stool in patients who exhibited no symptoms of respiratory system 2, 4. This indicates likely virus discharge without signs and symptoms of respiratory involvement, which makes it difficult to diagnose and combat CVOID-19.

Studies have shown that inflammatory cells effusion and lower lymphocyte are associated with severe respiratory syndrome in the process of COVID-19. We therefore hypothesized that lower circulating lymphocyte (CLC) might predict the initial GI involvement and virus discharge in stool. We surmise our results would assist clinicians to better combat and prevent COVID-19 dissemination.

## Materials and Methods

### Selection and Description of Participants

Clinical and laboratory data were retrospectively collected and analyzed. The patients complained of initial GI involvement, including vomiting, diarrhea, and respiratory symptoms including cough, chest pain and other extra-pulmonary symptoms, with or without fever, attending the Sixth People's Hospital of Wenzhou, and the Second Affiliated Hospital of Wenzhou Medical University, were screened by qRT-PCR on oropharyngeal swab for SARS-CoV-2. Diagnostic criteria for COVID-19 is based on the CDCP (China) Diagnosis and Treatment of COVID-19 5. All the patients of positive results were enrolled. The exclusion criterium is intaking contaminated food and water ahead of admission. Data of serial stool examinations for SARS-CoV-2 by qRT-PCR were collected until it remained negative.

### Blood Routine Test and Serologic Test

Fasting venous blood samples were collected and analyzed by standard method in the clinical laboratory center. Blood routine test was performed to count the blood cells and white blood cell classification, to measure C-reactive protein and hemoglobin concentration in the Mindray BC-5390 system (Shenzhen, China). The biochemical tests were performed in the VITROS 5600 Integrated Immunodiagnostic System (VITROS 5600, Johnson, New Jersey, U.S.A.), including albumin, total protein, lactic dehydrogenase, creatine kinase, alanine aminotransferase, aspartate transaminase, total bilirubin.

### Inspection of Virus Discharge in Stool

A ReadyPrep Prot RNA Extract Kit (Sol/Insol, BioRad) was used to extract total RNA from 200 μl of stool supernatant, following the manufacturers' instructions as mentioned elsewhere. RNA was eluted in 50μl of elution buffer and immediately used as the template for RT-PCR detecting SARS-CoV-2, which could be found in previous studies. Briefly, RNA template was added in the qPCR system using HiScript II One Step qRT-PCR SYBR Green Kit (Vazyme Biotech Co., Ltd). The 20μl qPCR reaction system contained 10μl 2× One Step SYBR Green Mix, 1μl One Step SYBR Green Enzyme Mix, 0.4μl 50× ROX Refernce Dye, 0.4μl of each primer (10μM) and 2μl RNA. The following program was performed for amplification: 50 ºC for 3 min, 95 ºC for 30 s followed by 40 cycles consisted of 95 ºC for 10 s, 60 ºC for 30 s, and a default melting curve step in an ABI 7500 machine 6.

### Statistics

Statistical data analysis was performed by using SPSS 25.0 (IBM, New York, U.S.A.). Data were presented as means ± SD and percentages for continuous and categorical variables, respectively. The categorial variables were compared among the patients applying one-way analysis of variance (ANOVA), Kruskal Wallis chi-square test, Fisher exact tests when appropriate, and continuous variables were compared among the patients applying Students' T test and ANOVA.

Multivariate linear regression model was applied to study the association between CLC and the risk of initial GI involvement. A dotted boxplot analysis was used to compare the difference in CLC distribution between the patients of COVID-19 with or without initial GI involvement. The difference in CLC was compared between the patients of positive and negative PCR results in stool. The receiver operation curve (ROC) was used to assess the predicting power of CLC and CRP on the virus discharge in stool. The CLC cutoff was calculated as sensitivity- (1 - specificity).

## Results

### Baseline Characteristics

Of the 76 enrolled COVID-19 patients, 24 patients (31.5%) presented with initial GI involvement on admission, including vomiting (7 cases, 29.2%), diarrhea (11 cases, 45.8%), and combined GI symptoms (6 cases, 25%). When compared with the COVID-19 patients without initial GI involvement (WT) on admission, those with initial GI involvement (WH) showed significantly faster heart rate (p = 0.033), higher proportion of which exhibiting fatigue (p = 0.009) and dyspnea (p = 0.003) (Table 1).

As shown in Table 1, no significant difference in age were found between patients of WT and those of WH. Compared to the WT group, significantly lower levels of CLC (p = 0.004) was found in WH group, while no differences were found in the circulating neutrophile count and plasma C reactive protein (CRP) levels between the two groups. There was also no significant difference in positive rate of qRT-PCR on stool detecting SARS-CoV-2 between patients of WH and WT.

### Association between CLC and viral discharge in stool & Initial GI Involvement

The precise association between CLC and initial GI involvement was analyzed using multiple logistic regression model (Table 2). Using 1^st^ tertile as reference, the odds ratio of initial GI involvement in the crude model for 2^nd^ and 3^rd^ tertiles decreased by nearly 74% (OR = 0.26 [95% CI: 0.07, 0.96]; p = 0.041) and nearly 85% (OR = 0.15 [95% CI: 0.04, 0.52]; p = 0.003), respectively. When adjusted for likely confounders, including gender, age, CRP, albumin globulin ratio, hypertension and dyspnea, similar association between CLC level and risk of initial GI involvement remained for 2^nd^ teritile (OR = 0.25 [95% CI: 0.07, 0.98]; p = 0.047) and 3^rd^ tertile (OR = 0.17 [95% CI: 0.05, 0.63]; p = 0.008) when compared to the 1^st^ tertile.

**Fig 1:**
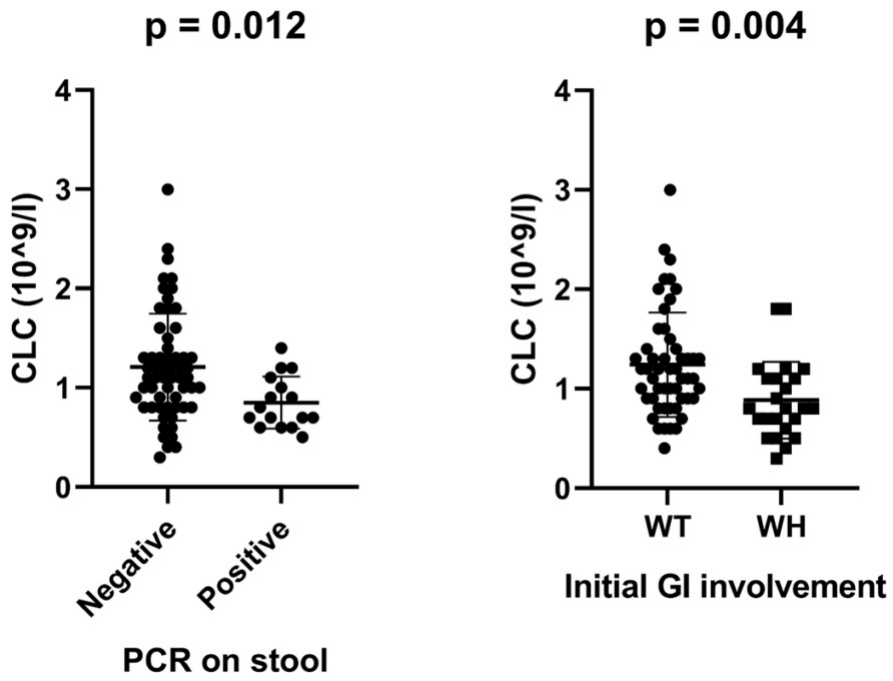
Dotted boxplot analysis of association between CLC and viral discharge in stool & Initial GI Involvement.

A dotted boxplot was used to illustrate the associations of CLC level with viral discharge and initial GI involvement of COVID-19 (Fig. 1). CLC level was found significantly lower in WH than that of WT group (p = 0.004). Moreover, significantly lower levels of CLC were found in those who tested positive by PCR via stool sample during the course of COVID-19 (Fig. 1).

### Predictive Power of CLC on Virus Discharge in Stool

**Fig 2:**
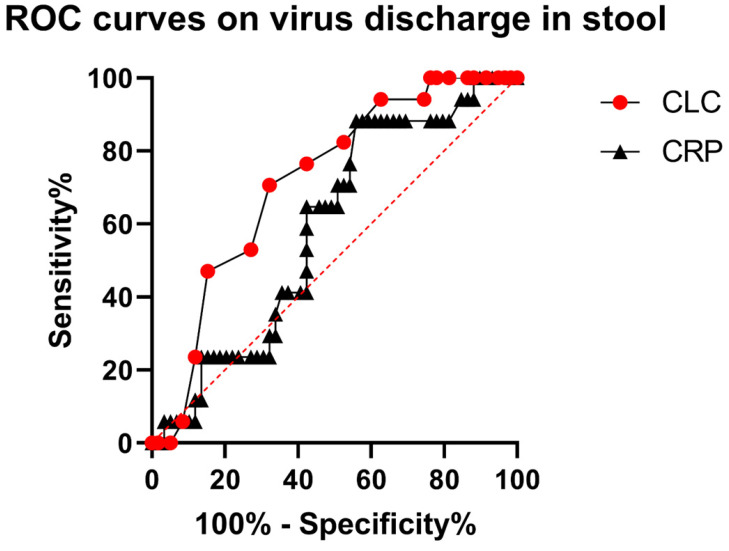
ROC curves of CLC and CRP.

ROC was used to assess the predicting power of CLC and CRP to assess virus discharge in the stool (Fig. 2). The area under curve of CLC is 0.72 (95% CI: 0.59, 0.85, p = 0.007), while that of CRP is 0.59 (95% CI: 0.45, 0.73, p =0.267). The cutoff value for CLC is at 0.95 × 10^9/l (sensitivity = 68.75%, specificity = 67.8%).

## Discussion

While concentrated efforts by the WHO and local governments continued to management and control this highly contagious and deadly disease, more than 600,000 confirmed death worldwide have been confirmed, and such number is expected to increase in the coming months. Therefore, it is crucial that more in-depth knowledge of COVID-19 should be gained.

Though SARS-CoV-2 was mainly identified as pulmonary pathogen, and therefore, the GI symptoms and continual viral shedding in stool are often overlooked, which in turn, might facilitate fecal-oral transmission of SARS-CoV-2. Emerging researches have shown that the virus nucleus could be detected in stool in the disease process, indicating virus discharge via GI tract yet the underlying mechanism is vastly unknown 2, 7, 8. It is also worrisome that no reliable and convenient biomarker was available to predict the risk of initial GI involvement and to predict virus discharge in stool of the COVID-19 patient.

### Correlation between CLC and Initial GI Involvement

In this retrospective observational study, a negative correlation between CLC and initial GI involvement was found, independent of age, gender, CRP, albumin globulin ratio, hypertension, and dyspnea. It is established that the angiotensin-converting enzyme 2 receptor was responsible for SARA-CoV-2 adhesion and invasion, which widely exists in the organs as pulmonary and GI tract 9, 10. However, not all of the COVID-19 patients exhibited initial GI involvement.

When compared to the 1st tertile of CLC levels, the risk of initial GI involvement decreased dramatically in the 2^nd^ and 3^rd^ tertile, which suggested a protective role of CLC against initial GI involvement. The natural history of the SARS-CoV-2 is reportedly incubated initially in the pulmonary tract and GI tract, and then proceeded to clinical symptoms if the cellular immune system was overwhelmed, which is congruent to our findings*.* That is to say, the decline of CLC levels in the course of COVID-19 might signal a virus discharge via the GI tract. Our result was further supported by the findings in existing investigations. Jiang 11 *et al* found that T subset of lymphocyte count in peripheral blood is down regulated in the cases of severe COVID-19 and Troche 12 *et al* reported that SARS-CoV-19 has shown likelihood to invade the mucosal of GI system, leading to water retention in the GI lumen and viral discharge. Furthermore, we found that lower CLC levels are significantly associated with positive PCR results in stool (Fig. 1), indicating a positive role of CLC in preventing viral discharge via GI tract. More interestingly, we did not find a significant difference in positive testing by qRT-PCR for stool between WT and WH patients, indicating the probability in virus discharge was not influenced by the occurrence of initial GI symptoms.

Higher plasma CRP level has previously been reported as positively related to the severity of lung injury and COVID-19 severity 13, 14. However, in our study, no significant correlation between plasma CRP level and the risk of initial GI involvement were found (Table 1). This demonstrated, for the first time that, CLC is better than plasma CRP level for assessing the odds of initial GI involvement in COVID-19 patients, which should be made aware to physicians currently managing the disease.

### Predictive Power of CLC for Virus Discharge in Stool

No significant differences were found between WH and WT groups in the proportion of patients testing positive by qRT-PCR in stool samples, indicating the virus might discharge through GI tract regardless of presence of GI symptoms. Therefore, a reliable biomarker is needed to predict viral discharge in stool. The levels of CLC was found to be a likely candidate for predicting virus discharge via GI tact was analyzed using ROC curve. Finally, we observed a better performance in predicting virus discharge in stool by CLC, when it compared with plasma CRP (Fig. 2).

The major limitation of this study included the relatively small sample size from the two medical centers, which might lead to possible selection bias, while the major strengthen is that the research is the first study on the risk factors on initial GI involvement and virus discharge in stool. In addition, the Asian ethnicity and the lack of medical history in this cohort might influence the generalizability of our results in other ethnic groups. Therefore, it remains to be established whether the inverse relationship between either CLC and viral discharge or CLC and initial GI involvement in further validation studies.

## Conclusions

In conclusion, the results of this preliminary study suggest lower CLC means higher risk of virus discharge in stool of COVID-19 patients, and higher odds of initial GI involvement. More care and active monitoring for serum CLC levels are need to better evaluate the course of COVID-19 in patients who might be on the trajectory of recovery.

## Figures and Tables

**Table 1:** Baseline characteristics of included patients, stratified by initial GI involvement.

	WT (n = 52)	WH (n = 24)	*p*-value
**Demographics**			
Age (years)	44.3 ± 12.6	45.1 ± 13.4	0.807
Male (%)	24 (46.2)	10 (41.7)	0.715
Higest Temperature (degree centigrade)	38.0 ± 0.7	38.3 ± 0.5	0.095
Pulse per minute	90 ± 10	96 ± 12	0.033
Hypertension (%)	11 (21.15)	7 (29.17)	0.445
Fatigue (%)	20 (38.46)	17 (70.83)	0.009
**Respiratory Manifestation**			
Breath (times per minute) (%)			0.066
16	1 (1.92)	0 (0.00)	
17	1 (1.92)	0 (0.00)	
18	5 (9.62)	0 (0.00)	
19	1 (1.92)	4 (16.67)	
20	38 (73.08)	15 (62.5)	
21	2 (3.85)	1 (4.17)	
22	4 (7.69)	2 (8.33)	
23	0 (0.00)	2 (8.33)	
Dyspnea (%)	5 (9.62)	9 (37.5)	0.004
Cough (%)	32 (61.54)	18 (75)	0.250
**Laboratory Characteristics**			
White Blood Cell count (10^9/l)	4.57 ± 1.52	4.21 ± 1.41	0.335
Neutrophile Count (10^9/l)	2.87 ± 1.20	3.06 ± 1.27	0.534
Lymphocyte count (10^9/l)	1.24 ± 0.53	0.88 ± 0.38	0.004
Hemoglobulin (g/l)	137.79 ± 11.40	133.63 ± 15.74	0.195
Platelet count (10^9/l)	180.81 ± 64.66	164.13 ± 60.72	0.116
C Reactive Protein (mg/l)	16.96 ± 21.30	27.99 ± 25.41	0.052
Total Protein (g/l)	71.36 ± 5.85	70.12 ± 5.56	0.385
Globulin (g/l)	29.86 ± 5.24	29.46 ± 4.24	0.745
Albumin Globulin Ratio	1.43 ± 0.27	1.41 ± 0.25	0.719
Lactic dehydrogenase (U/L)	216.37 ± 70.32	248.13 ± 90.66	0.100
Creatinine (mmol/l)	69.50 ± 15.02	73.32 ± 18.55	0.330
Alanine Aminotranferase (U/L)	28.73 ± 26.19	33.00 ± 24.25	0.501
Aspartate transaminase (U/L)	31.65 ± 18.77	34.67 ± 27.62	0.579
Total Bilirubin (mmol/l)	15.32 ± 8.36	13.80 ± 8.65	0.470
qRT-PCR on stool (%)	10 (18.18)	7 (29.16)	0.334

Note: qRT-PCR: quantitative real-time Polymerase chain reaction; WT: Patients of COVID-19 presented without gastrointestinal tract affected on admission; WH: Patients of COVID-19 presented with gastrointestinal tract affected on admission. Data are presented as means ± SD, or n (%).

**Table 2:** Relationship between CLC and initial gastrointestinal involvement.

	Initial gastrointestinal involvement				
CLC	Crude Model		Adjusted Model I		Adjusted Model II
tertile	OR (95% CI)	p value	OR (95% CI)	p value	OR (95% CI) p value
1	1		1		1
2	0.26 (0.07, 0.95)	0.041	0.25 (0.07, 0.92)	0.038	0.25 (0.07, 0.98) 0.047
3	0.15 (0.04, 0.52)	0.003	0.14 (0.04, 0.52)	0.003	0.17 (0.05, 0.63) 0.008

Crude Model adjusted for None. Adjusted Model I adjusted for age, gender. Adjusted Model II adjusted for age, gender, CRP, albumin globulin ratio, hypertension, dyspnea. CLC Tertile 1 ≤ 0.88 * 10^9/l; 0.88 * 10^9/l < CLC Tertile 2 ≤ 1.2 * 10^9/l; CLC Tertile 3 > 1.2 * 10^9/l.
